# Supplementary material for: DCAF14 regulates CDT2 to promote SET8-dependent replication fork protection
Source: Life Sci Alliance. 2023 Nov 8;7(1):e202302230. doi: 10.26508/lsa.202302230 (PMC10631547; doi:10.26508/lsa.202302230)

Figure 1A

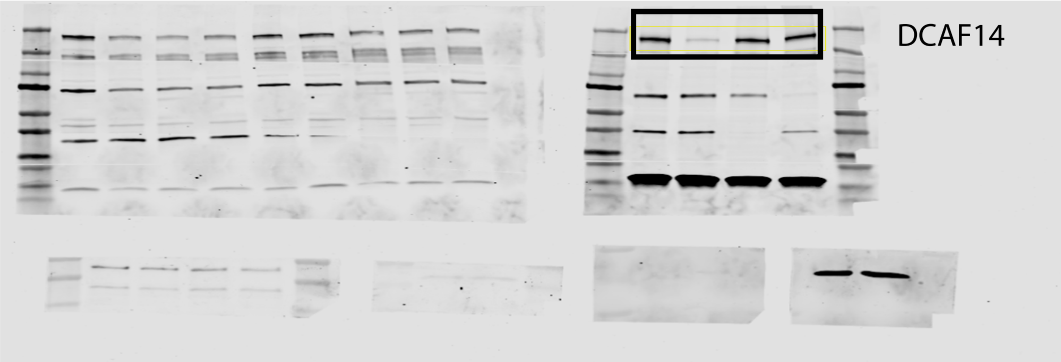

PCNA

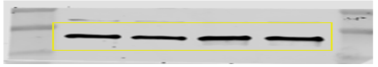

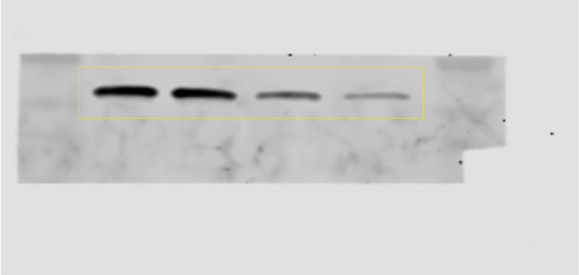

H3K4me3

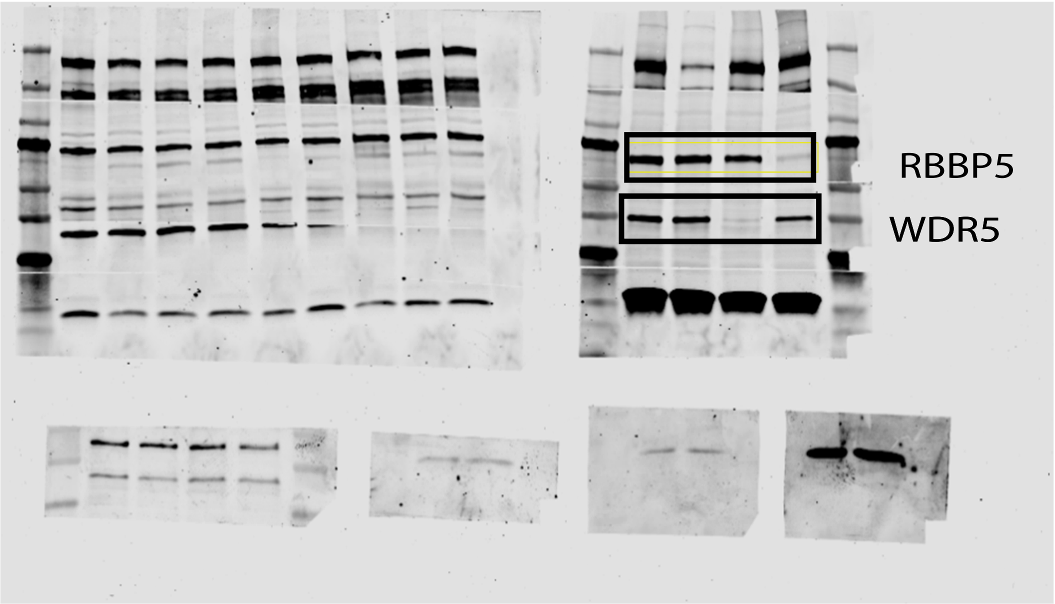

Figure 1B

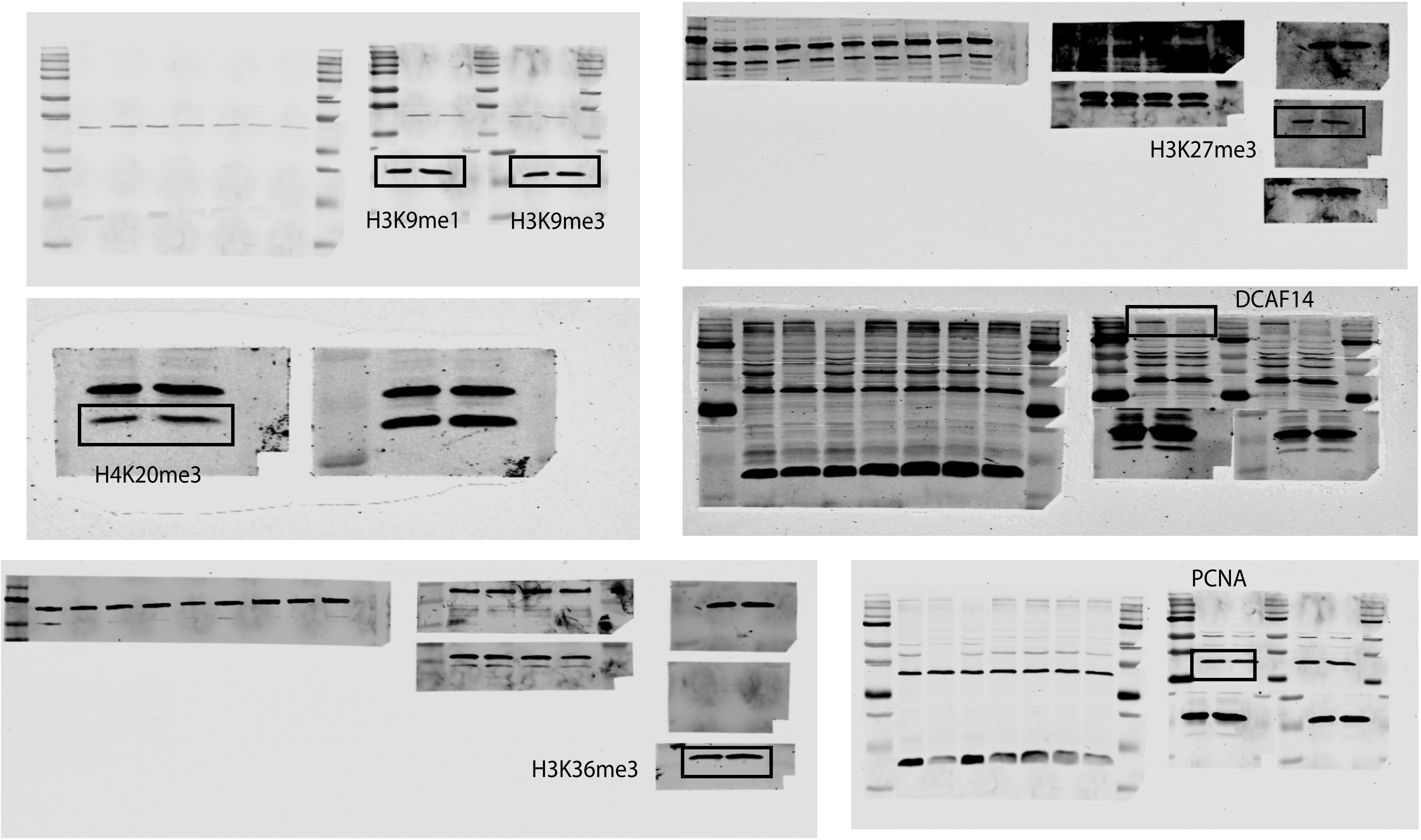

Figure 1C

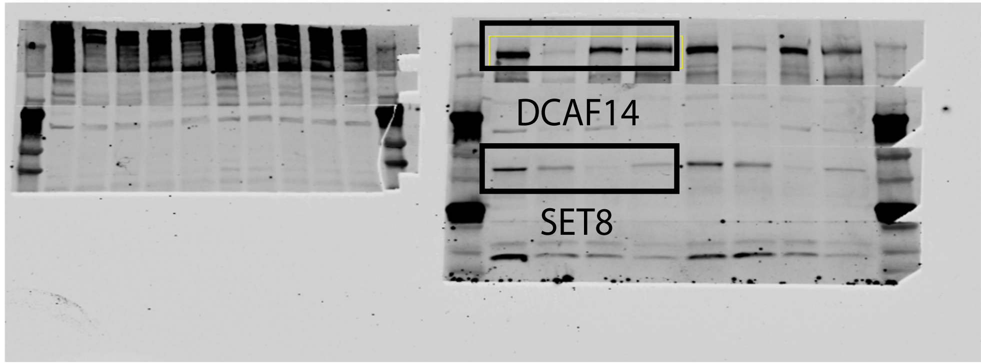

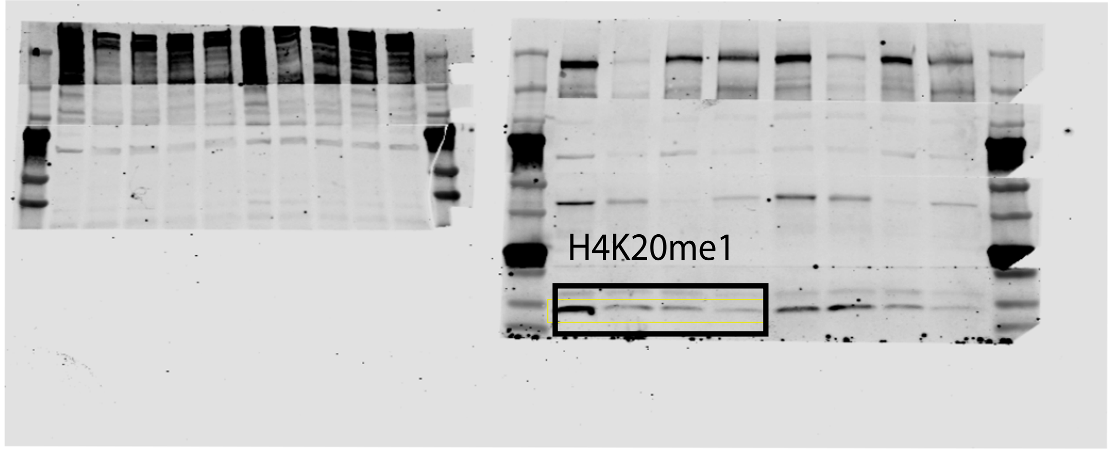

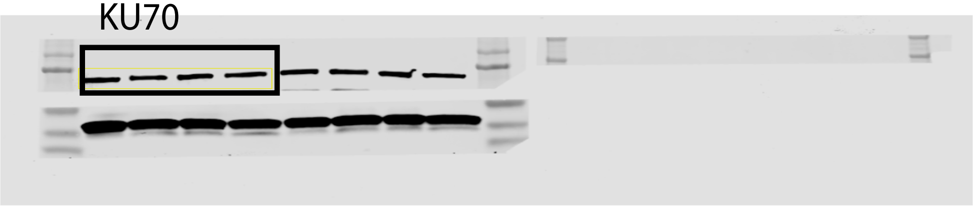

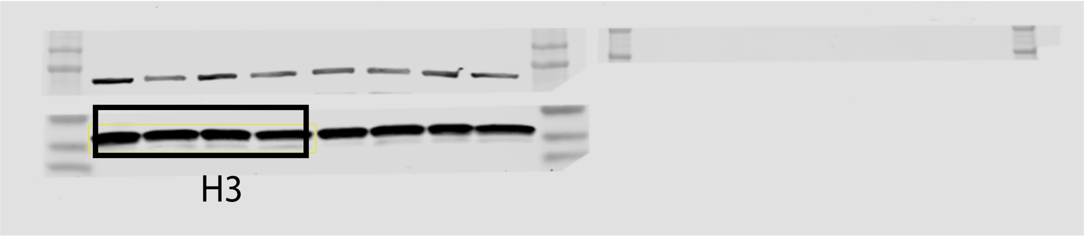

Figure 2A

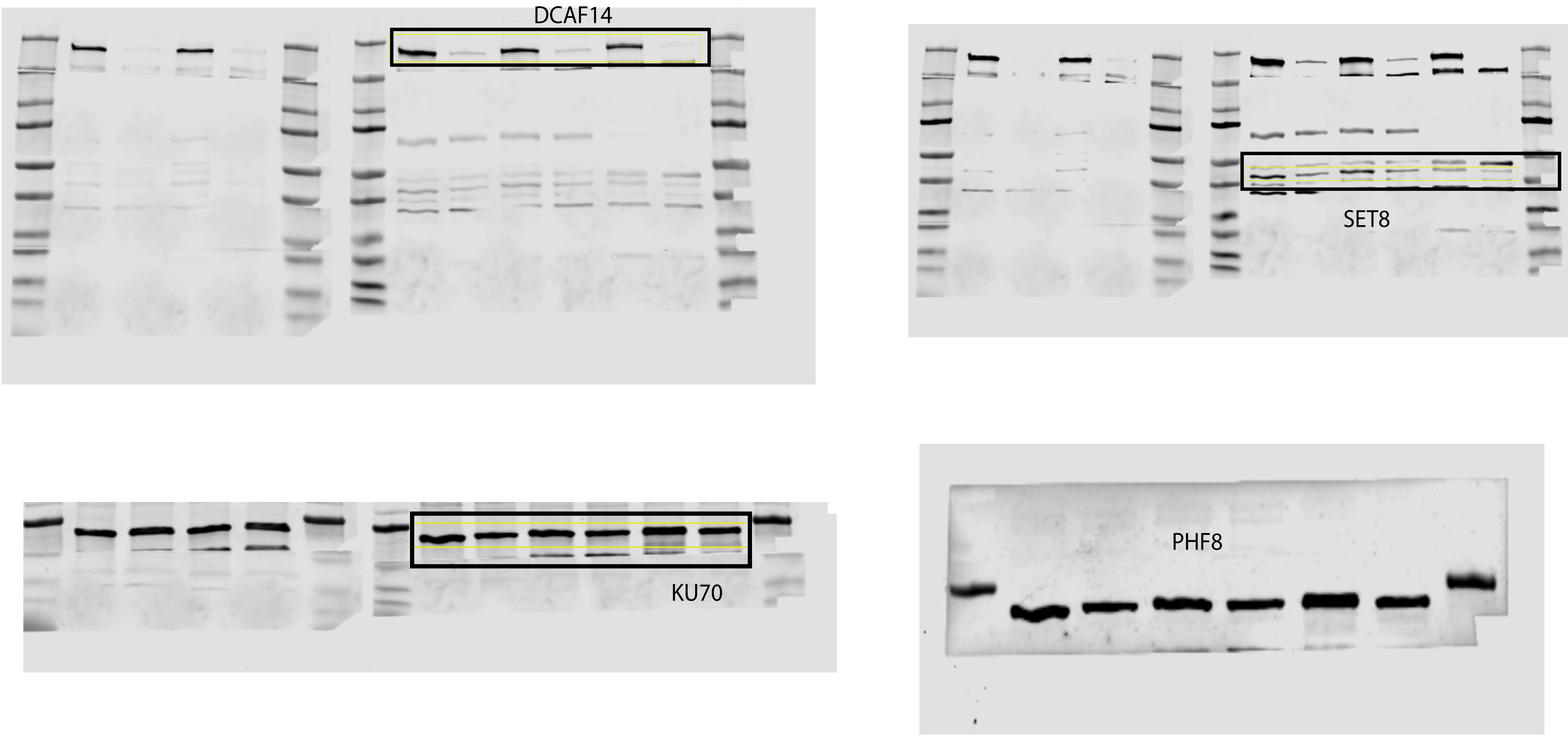

Figure 3A

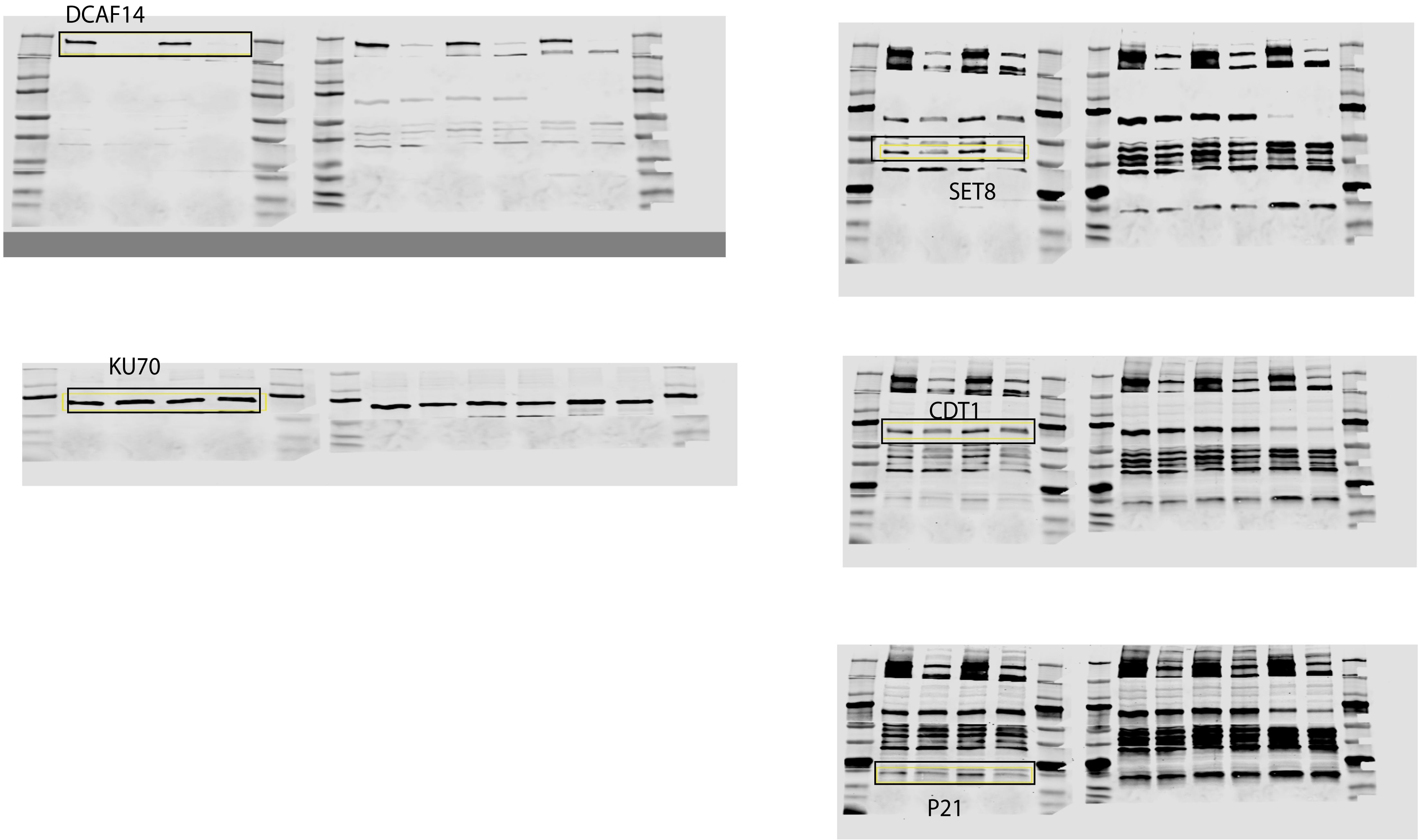

Figure 3F

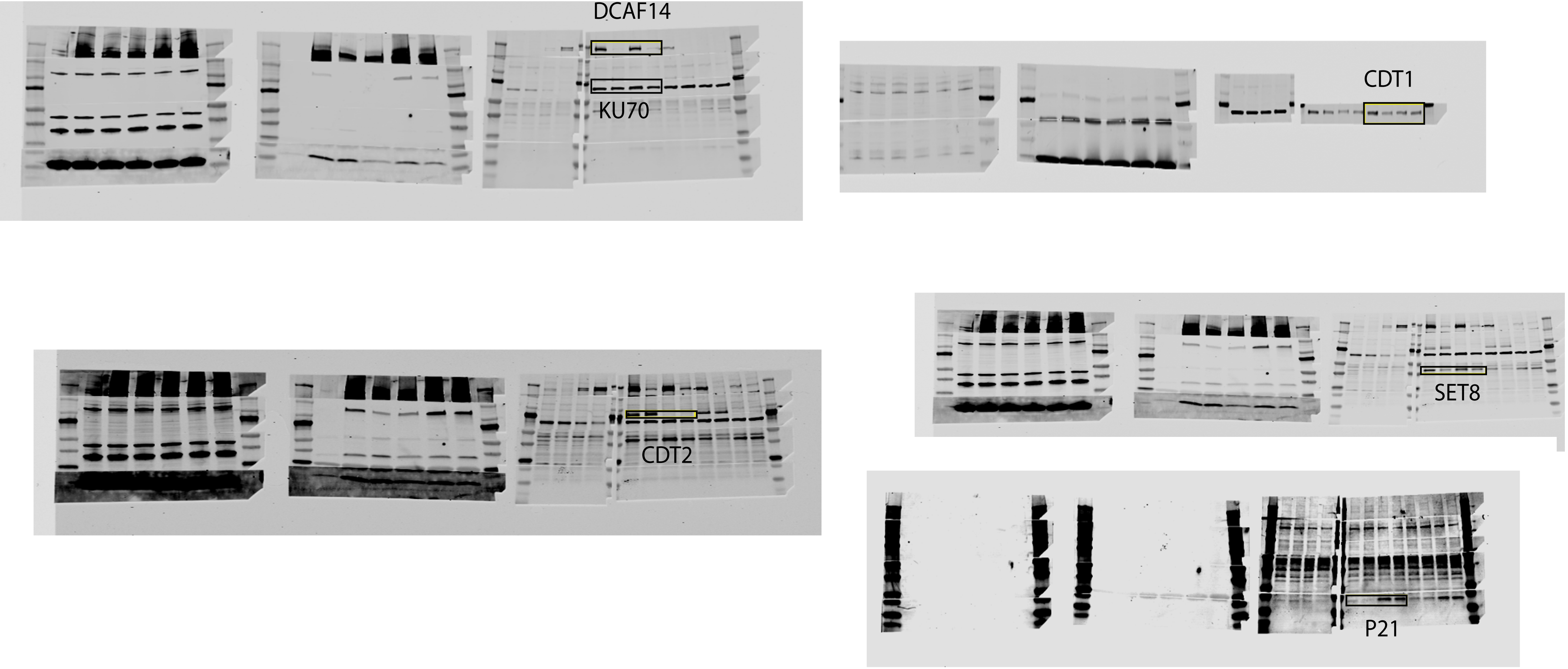

Figure S1B

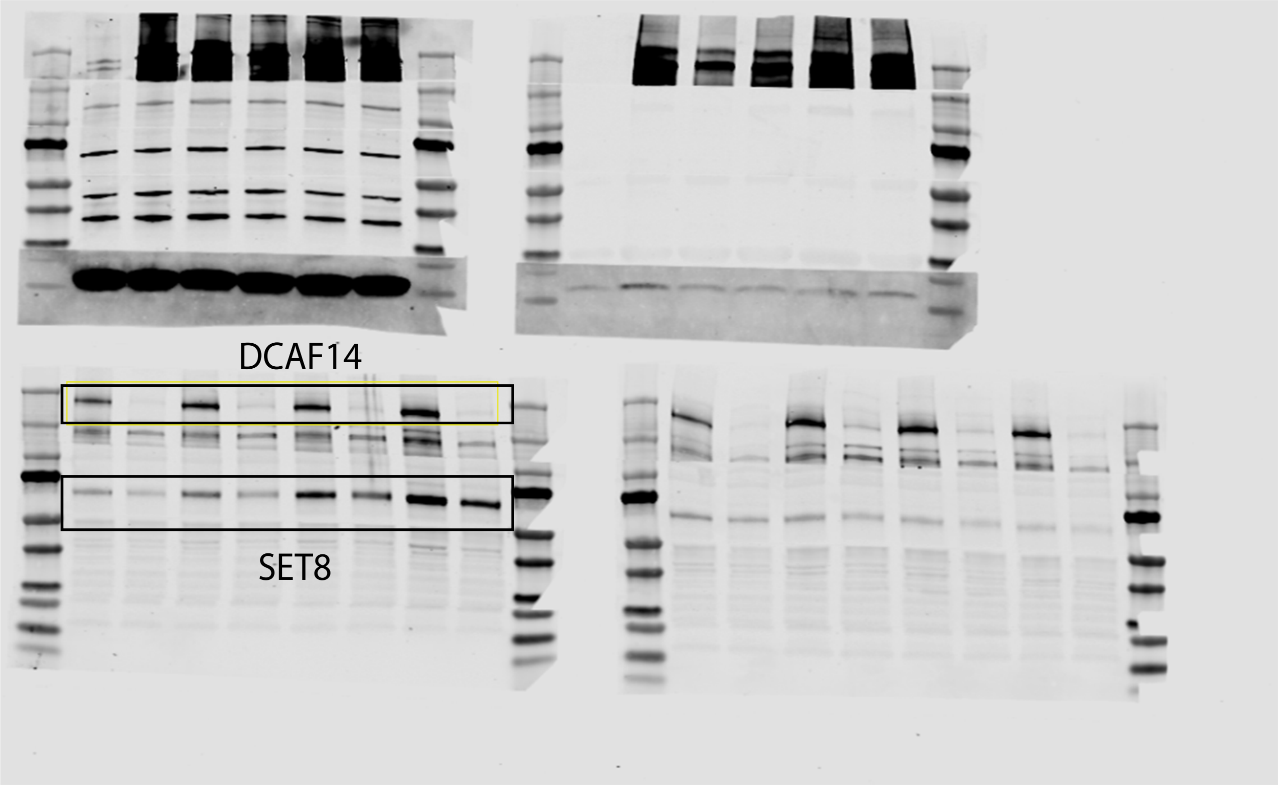

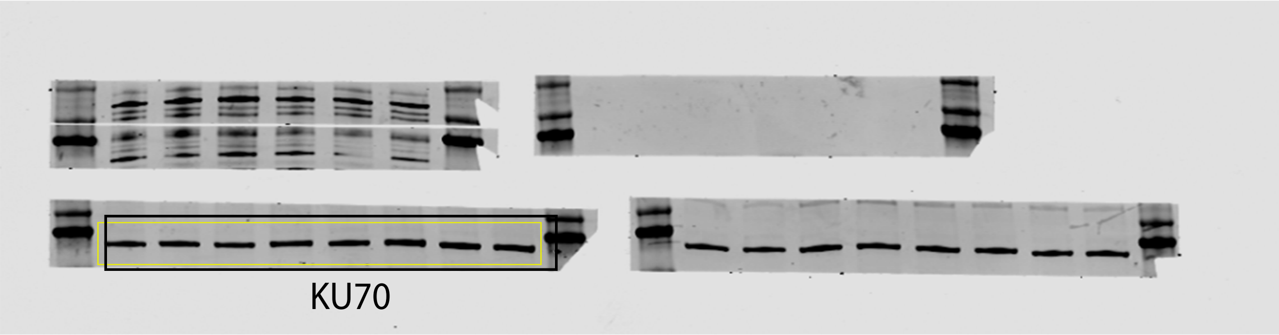

Figure S2D

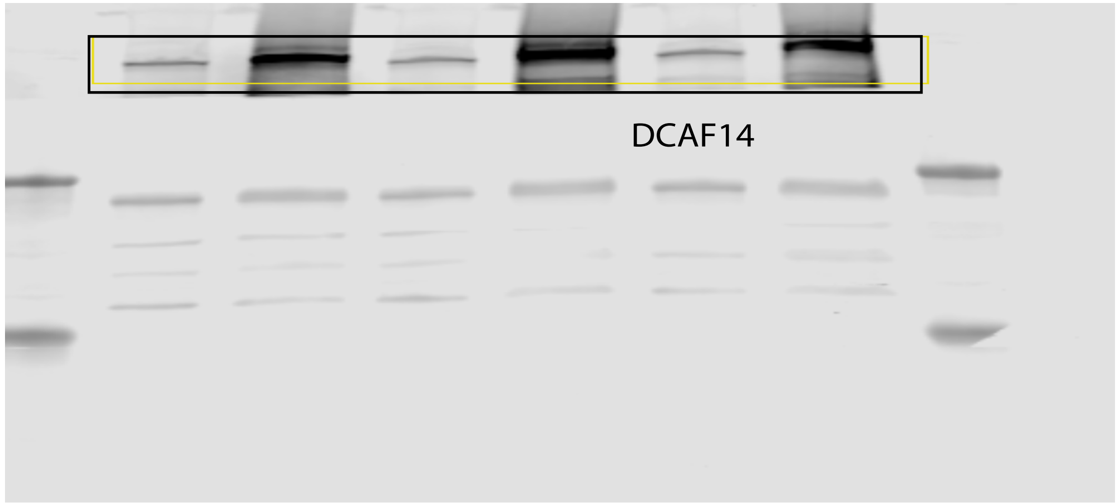

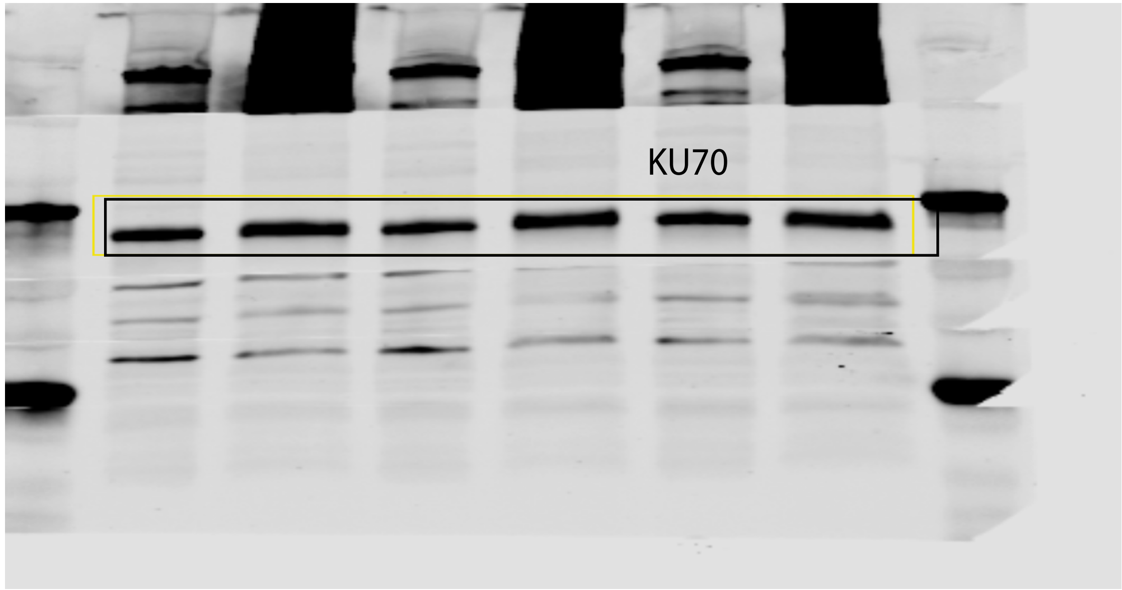

Figure S3A

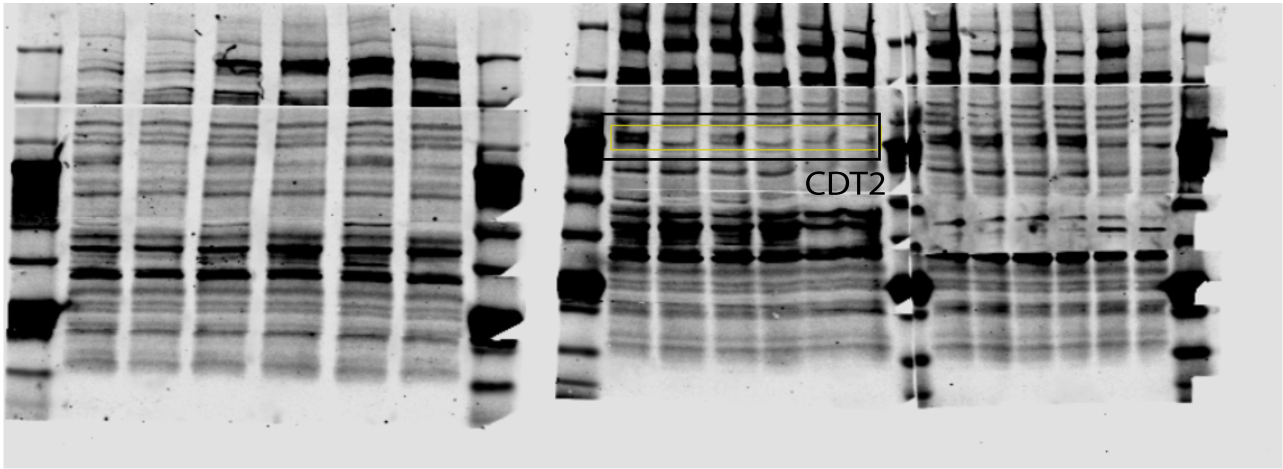

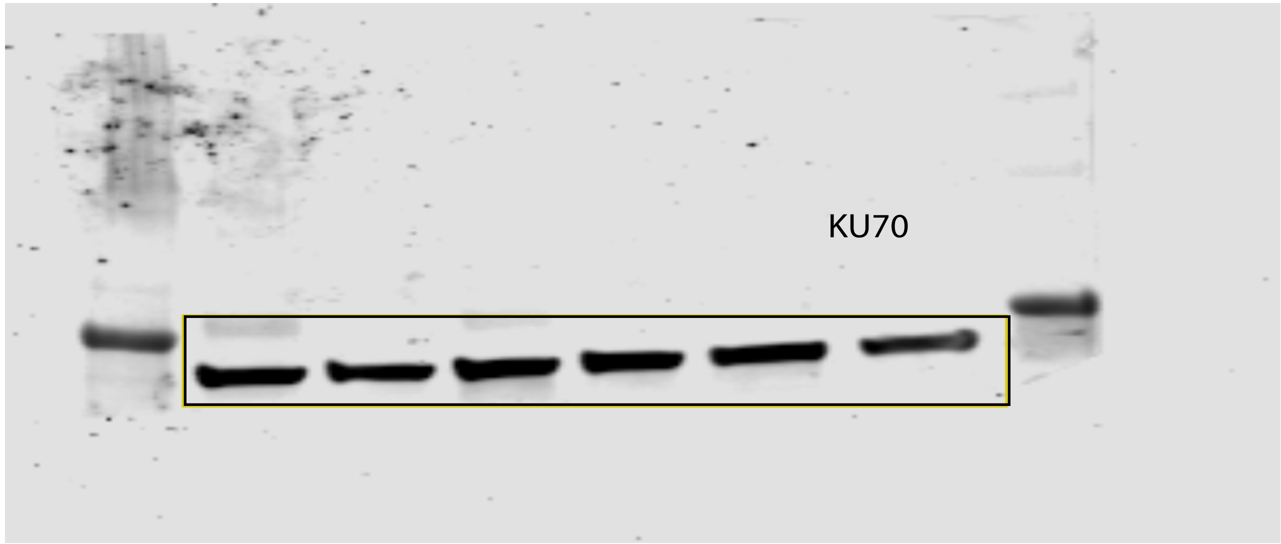

Figure S3C

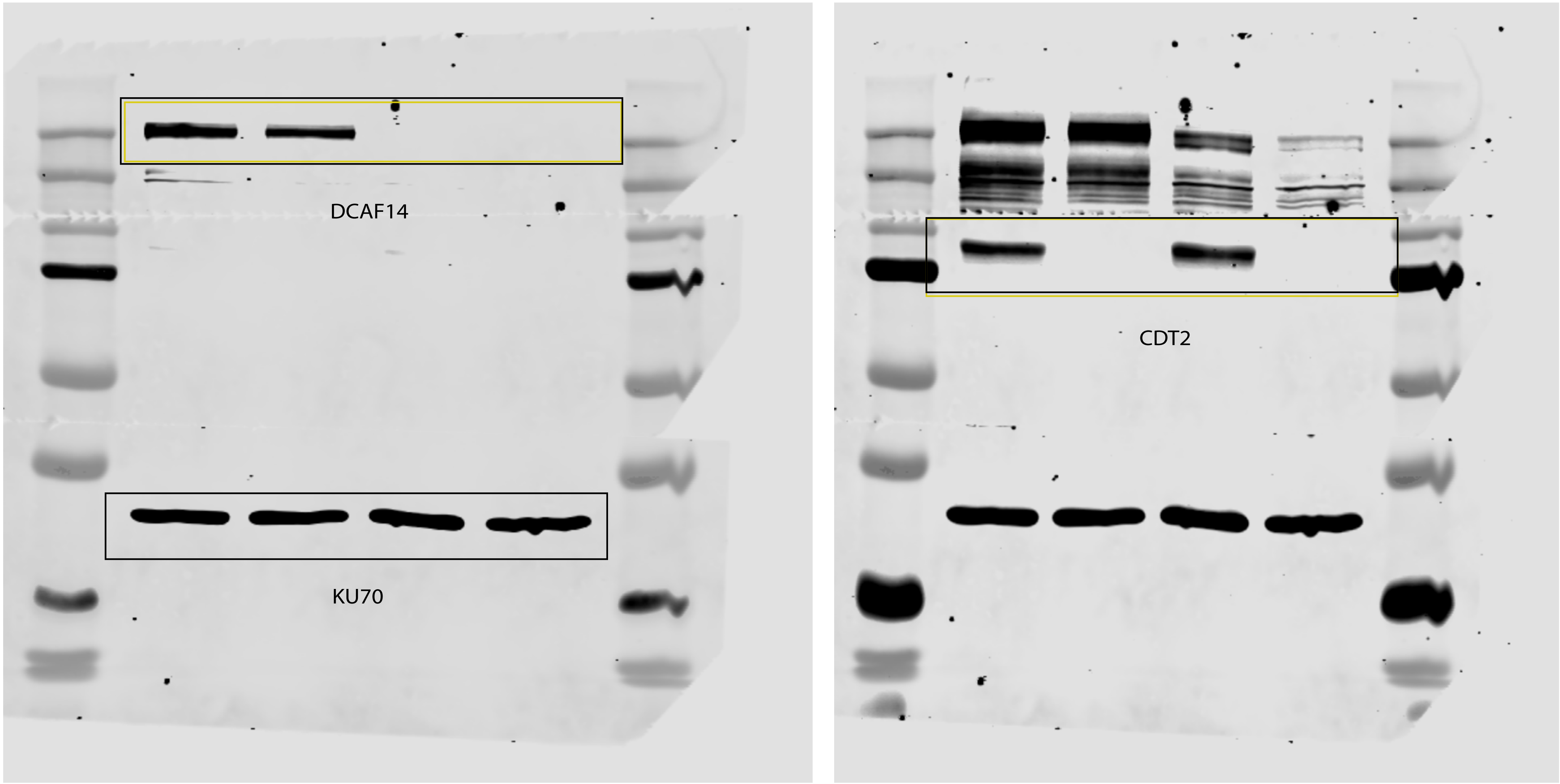

Figure S3E

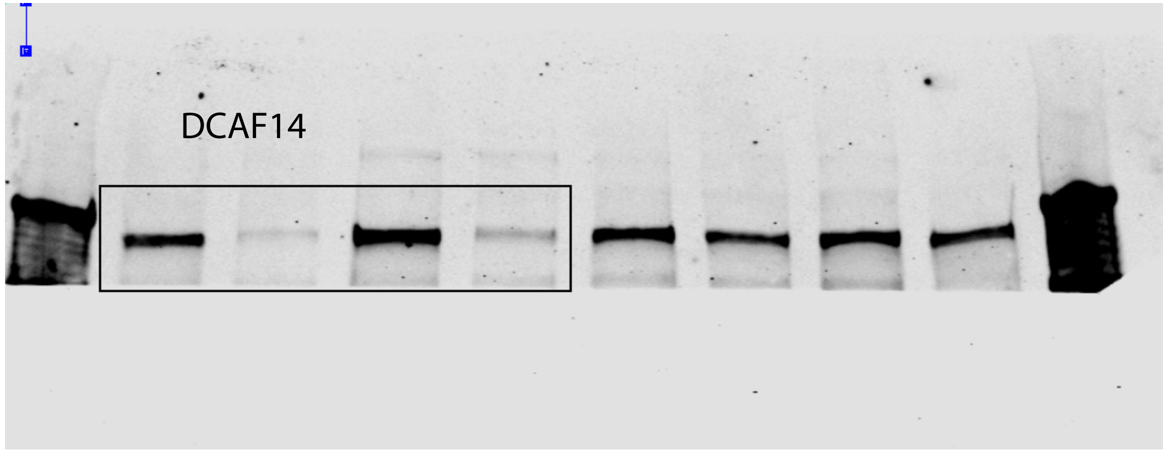

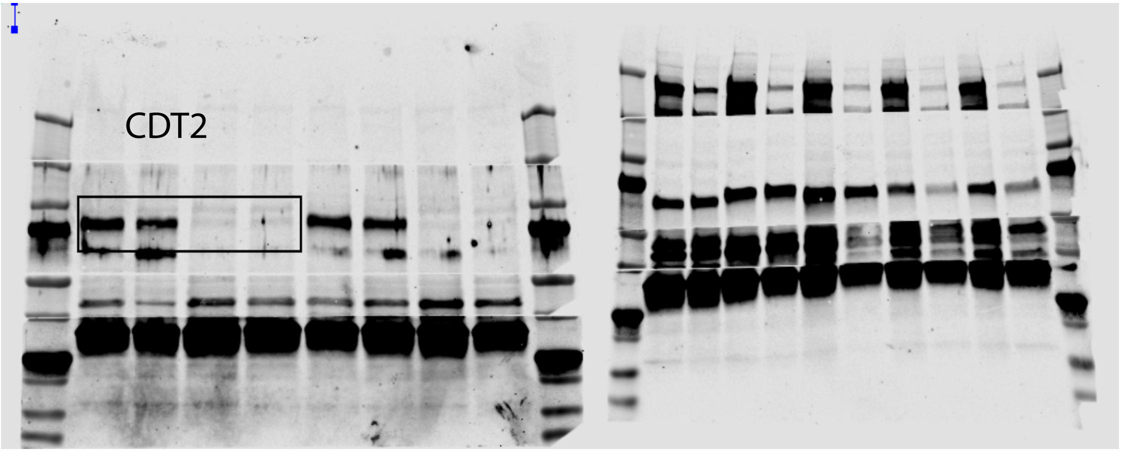

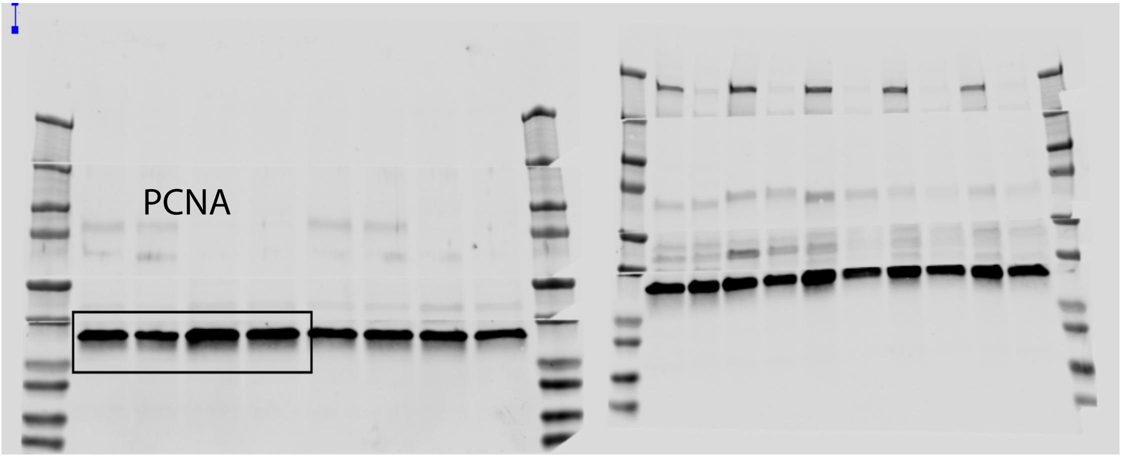

Figure S4A

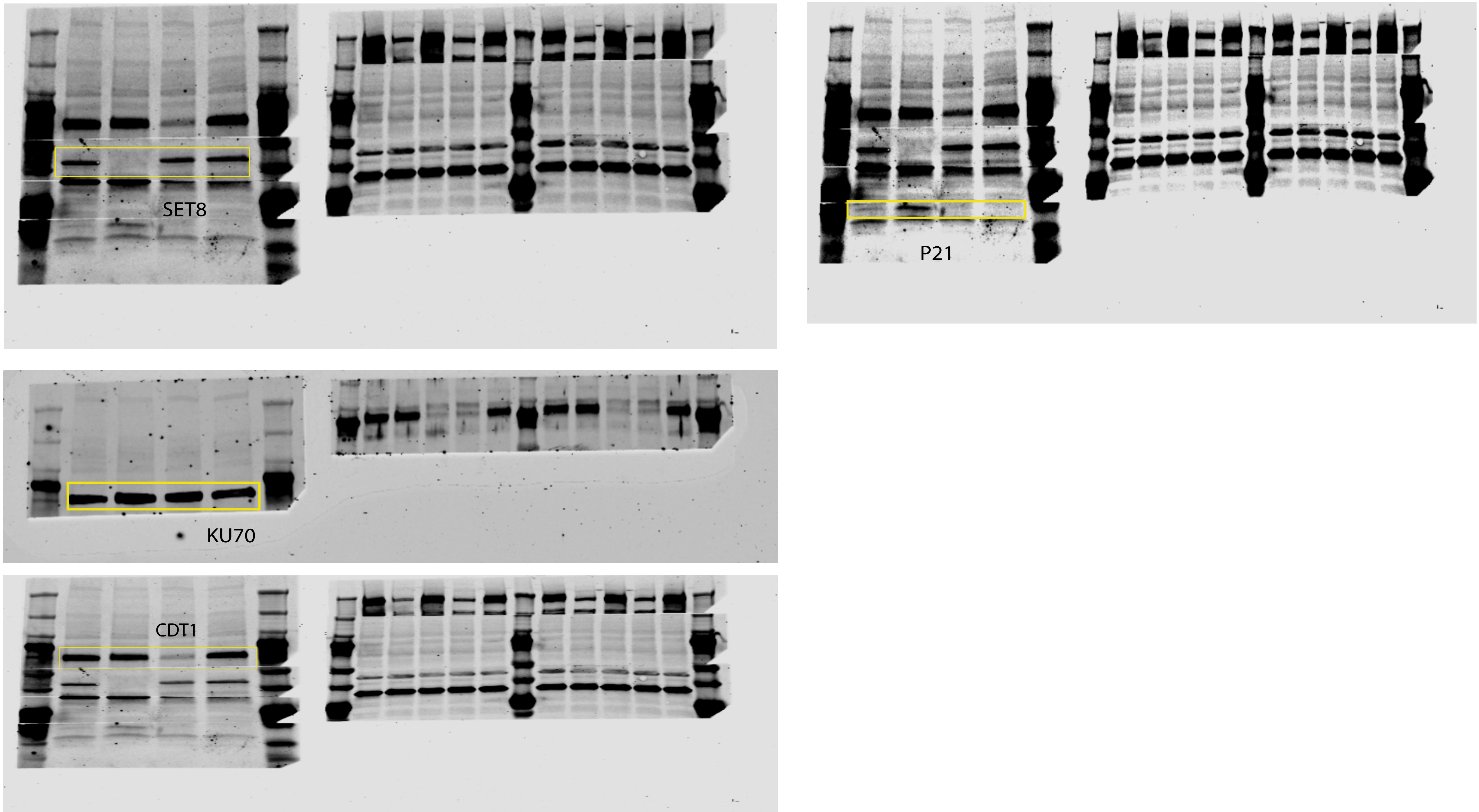

Figure S4C

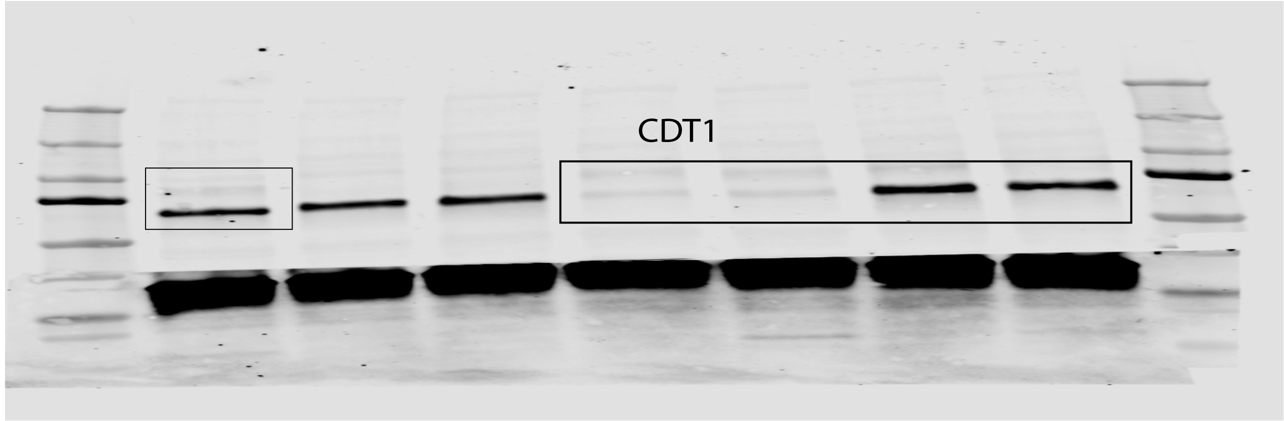

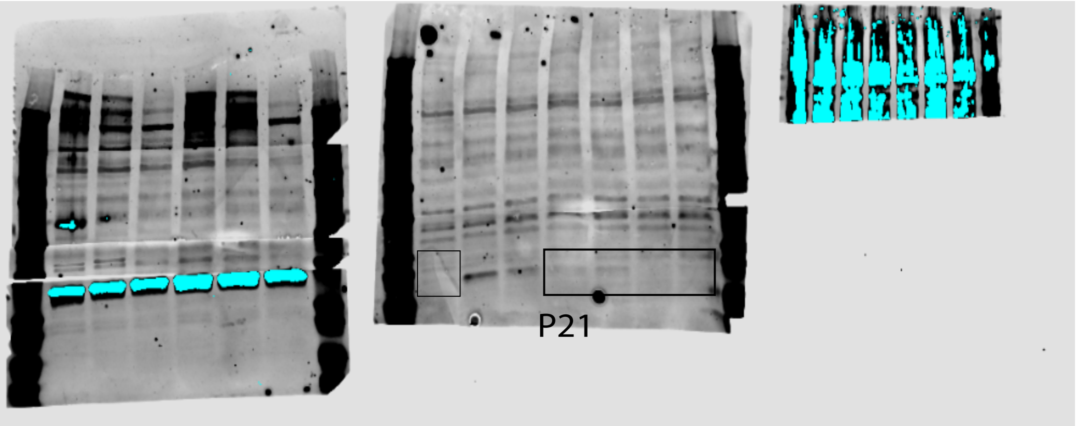

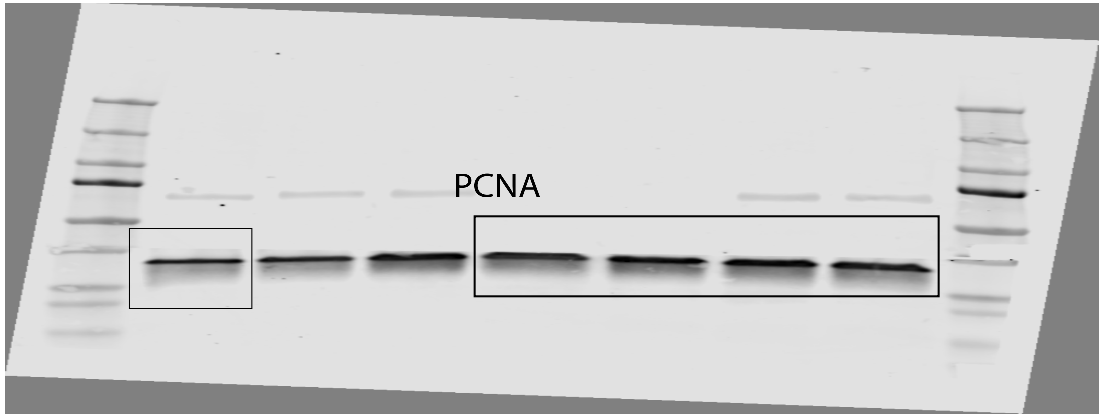

Figure S4E

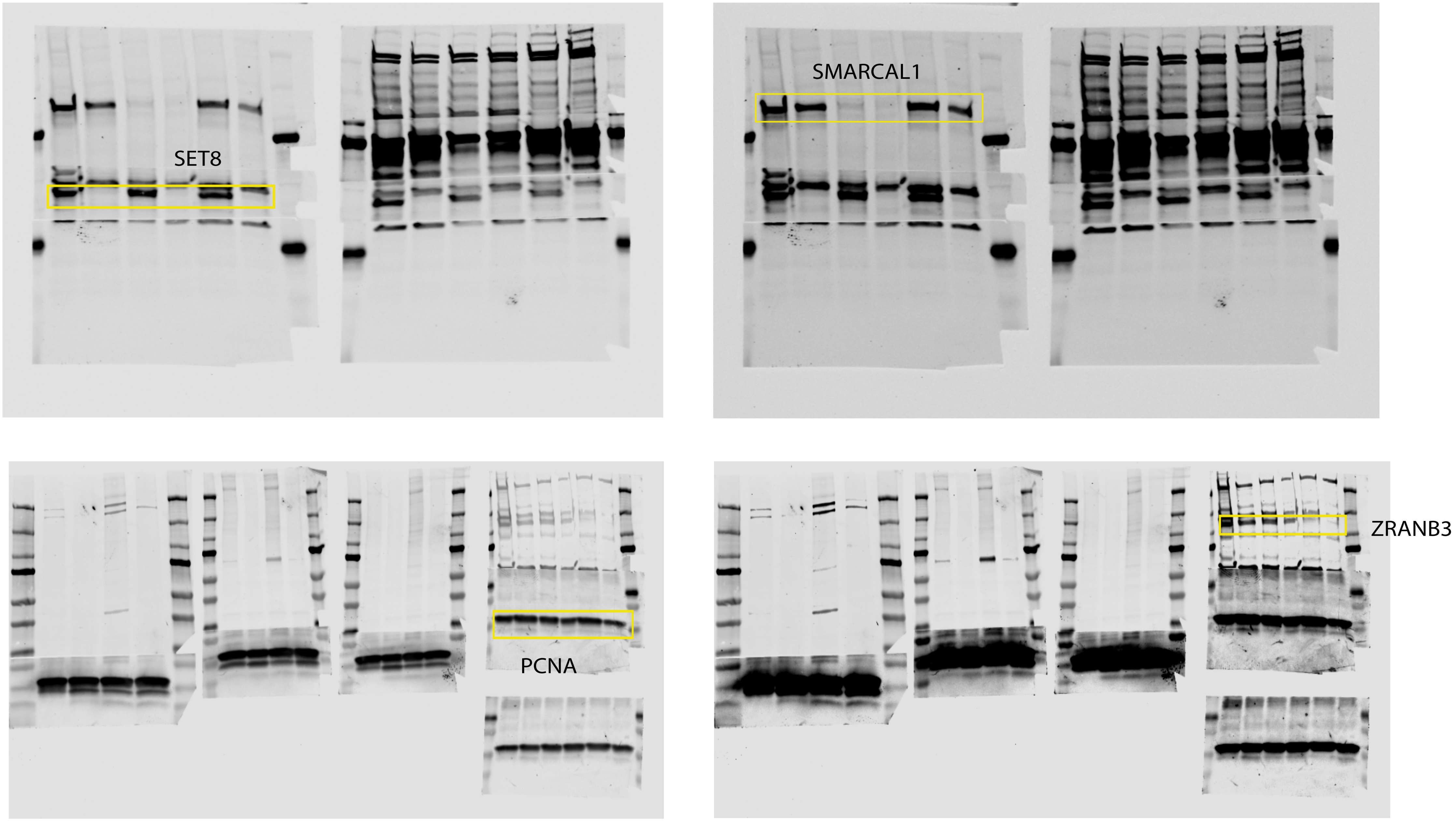

Figure S4G

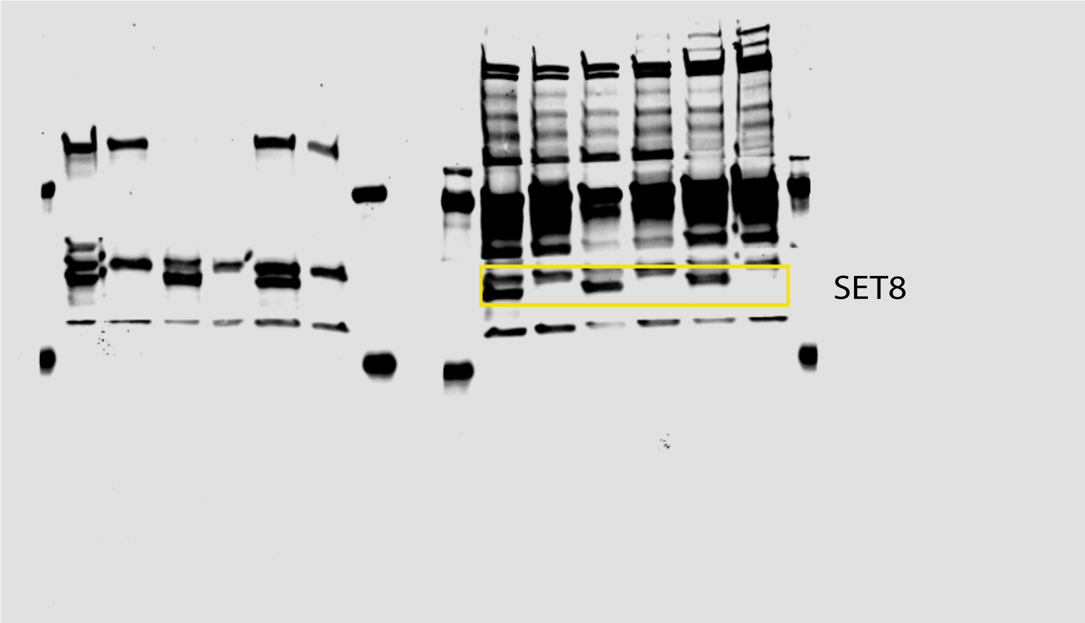

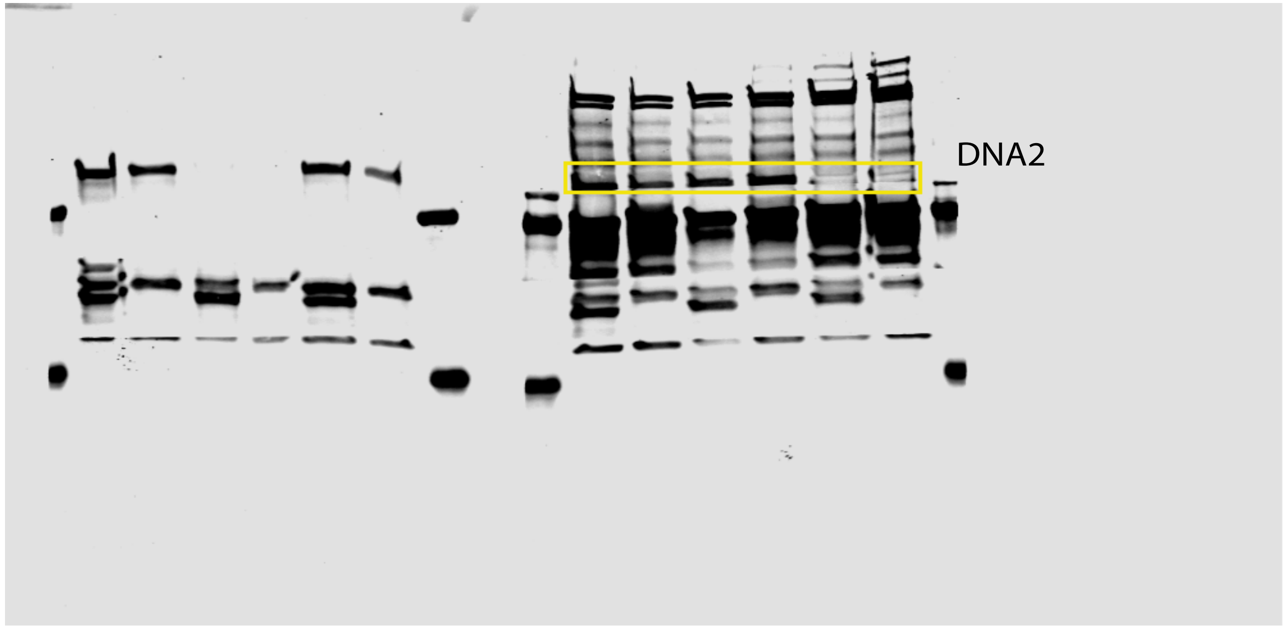

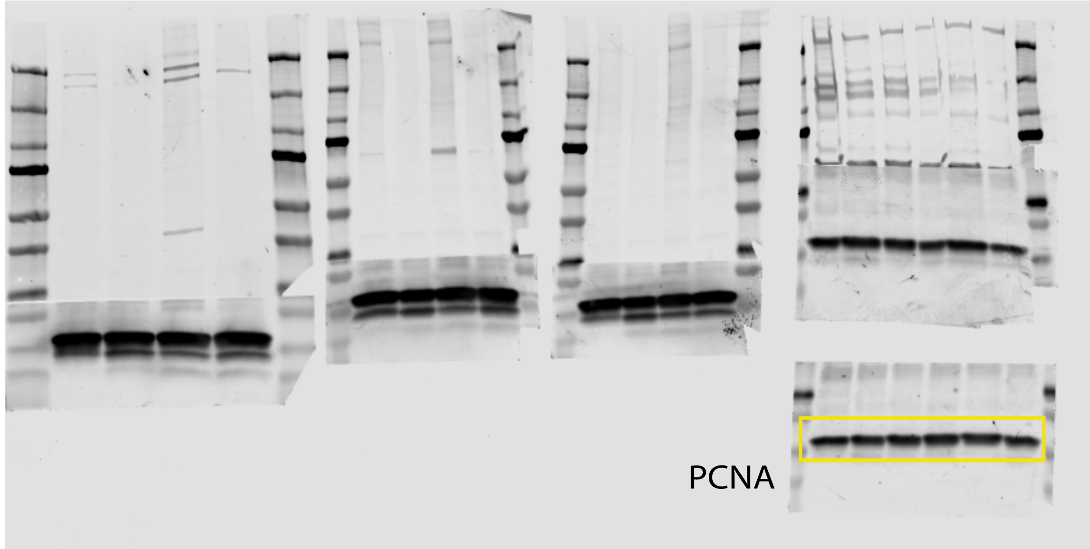

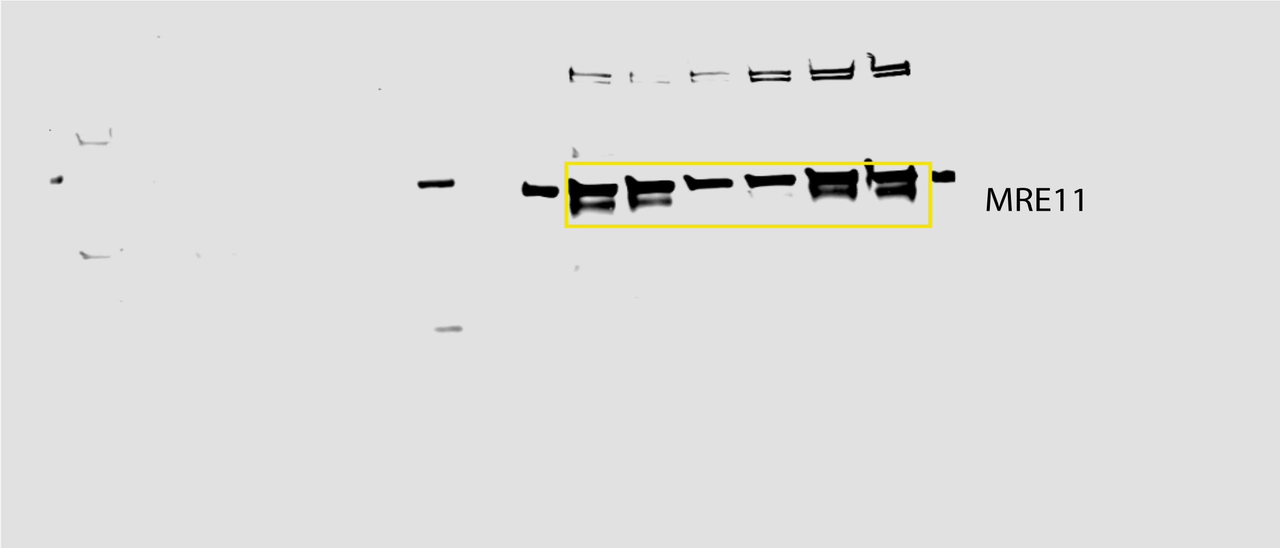

Figure S5A

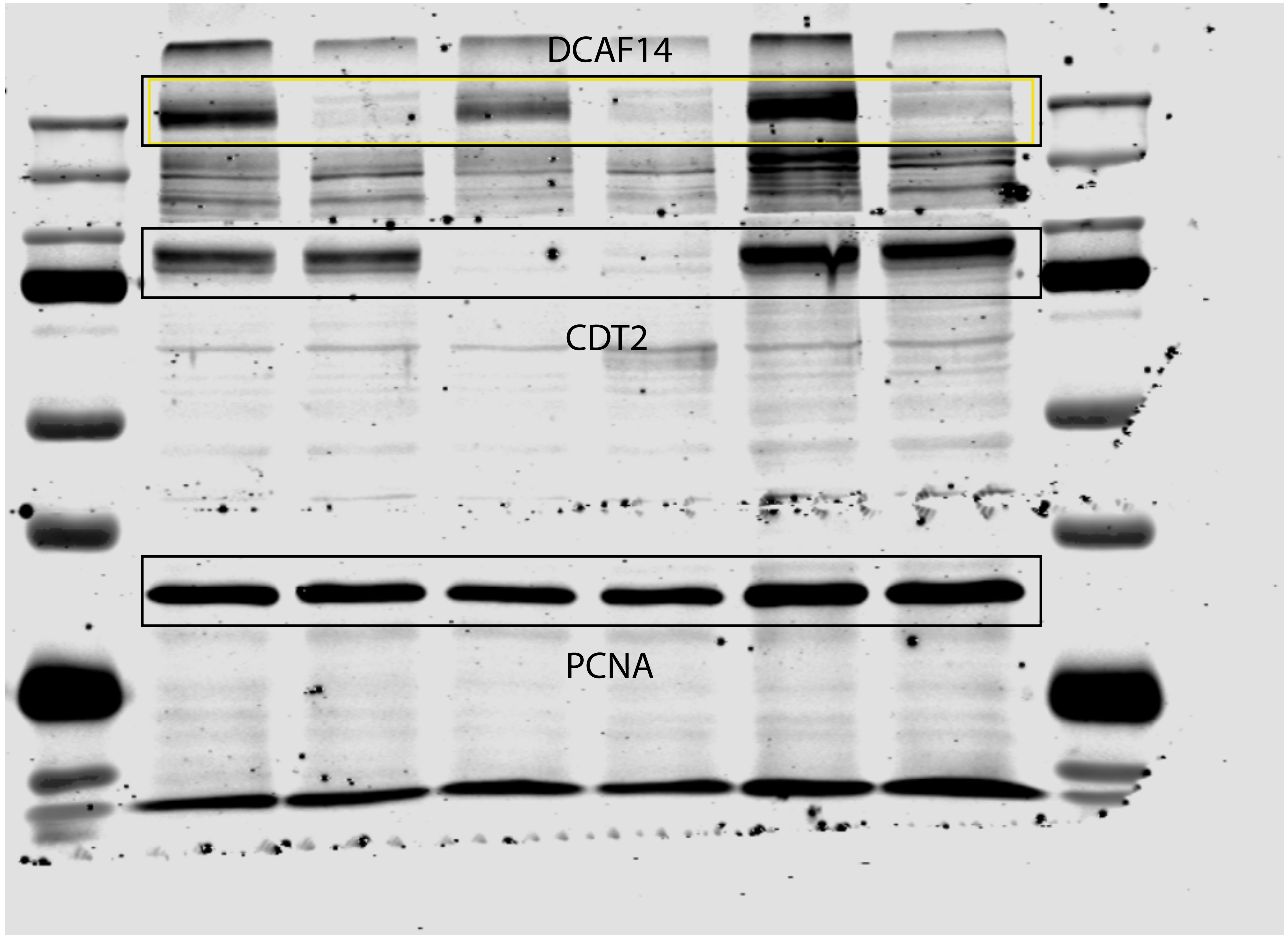

Figure S5B

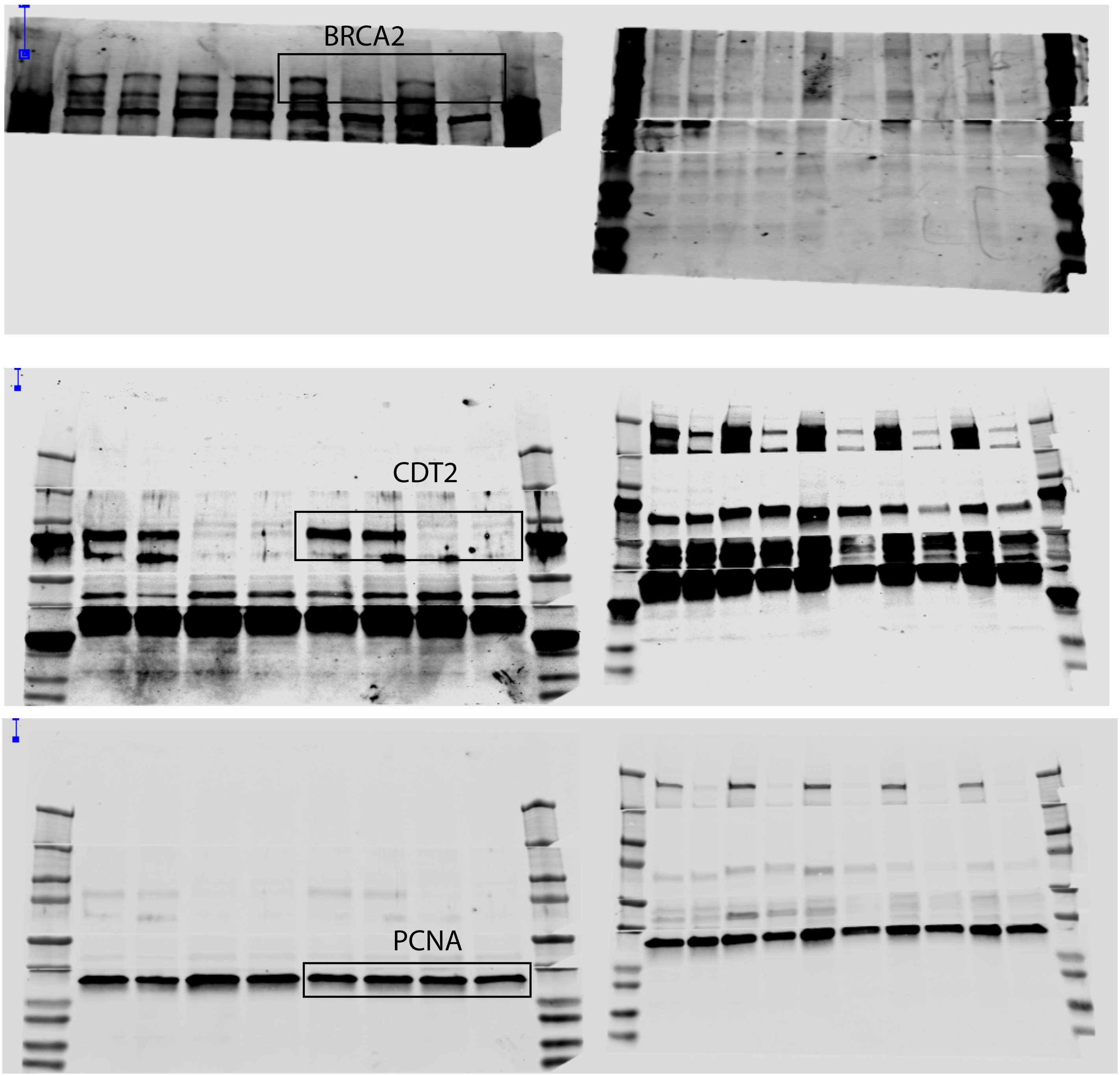

Figure S5D

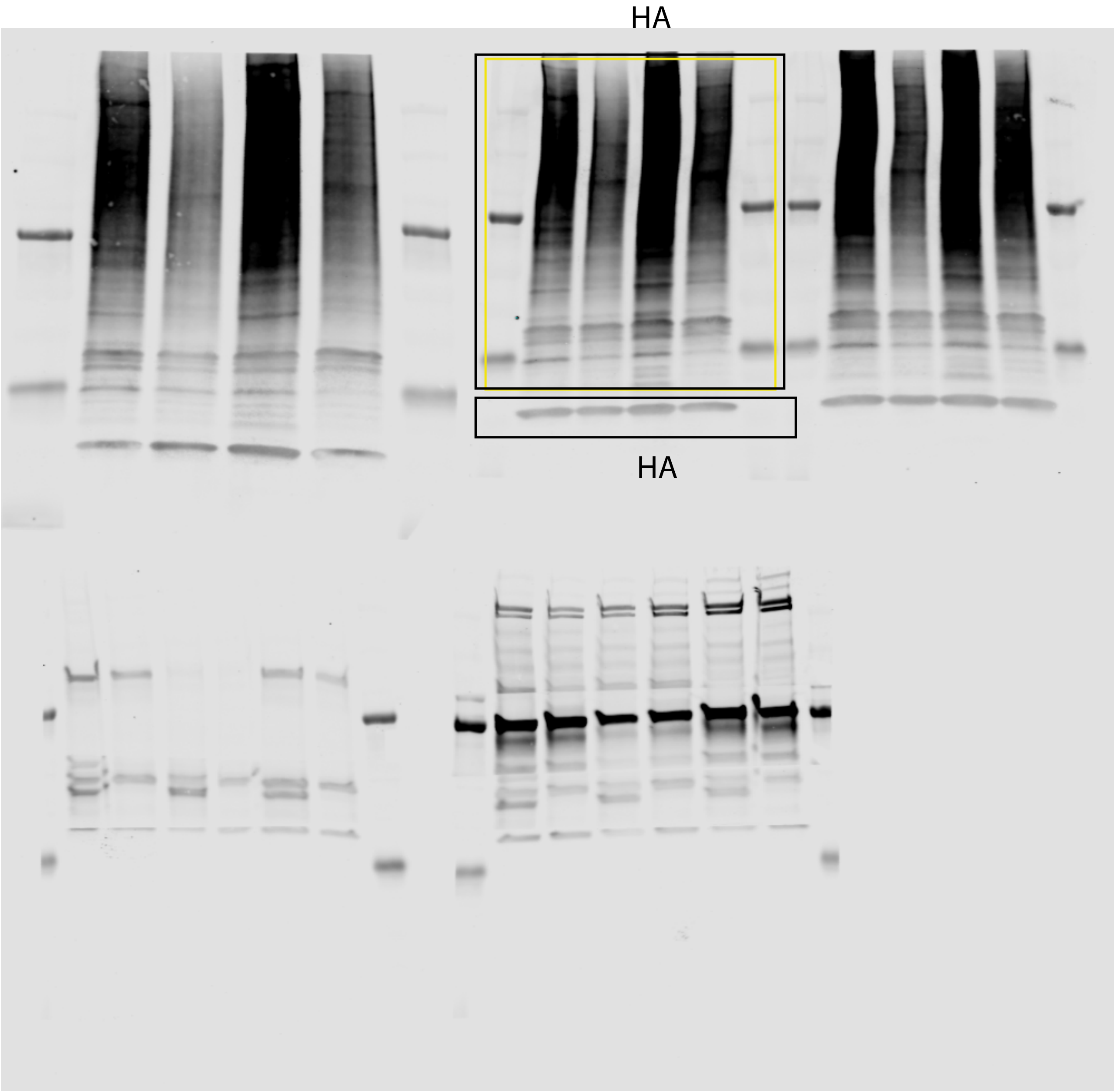

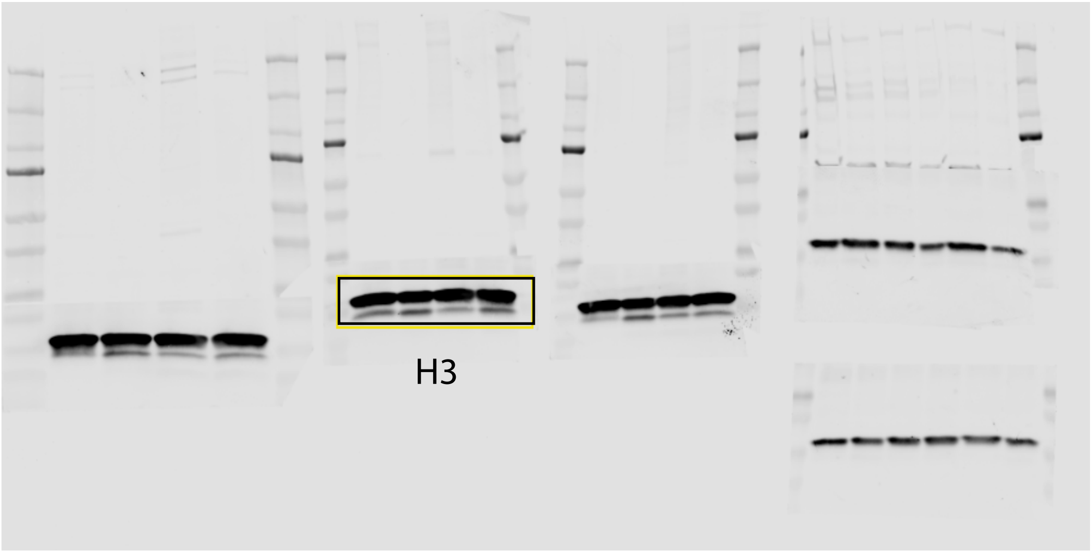

Supplement: Supplementary file 2 [file LSA-2023-02230_SdataF1.2_F2.2_F3.2_FS1.2_FS2.2_FS3.3_FS4.2_FS5.2.pdf]
