# Supplementary figures and images for: DCAF14 regulates CDT2 to promote SET8-dependent replication fork protection
Source: Life Sci Alliance. 2023 Nov 8;7(1):e202302230. doi: 10.26508/lsa.202302230 (PMC10631547; doi:10.26508/lsa.202302230)

Figure S3E

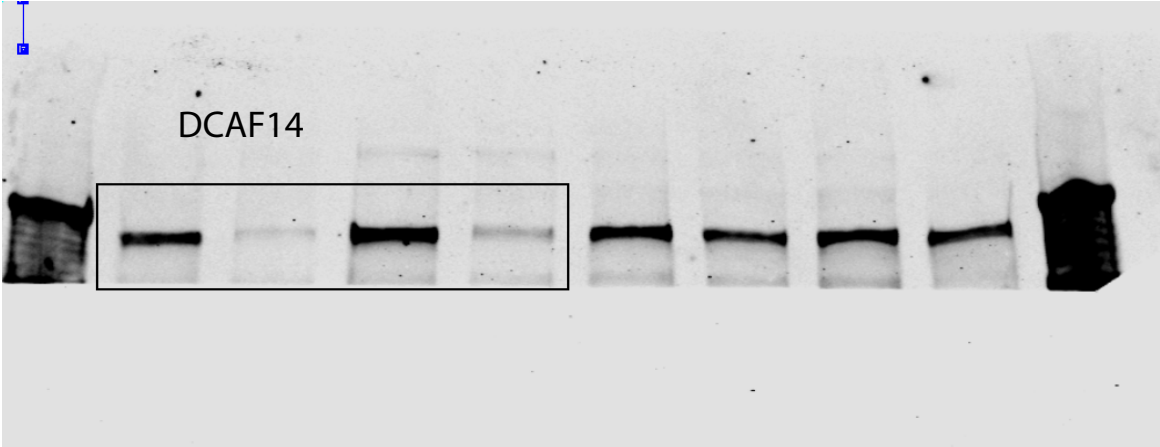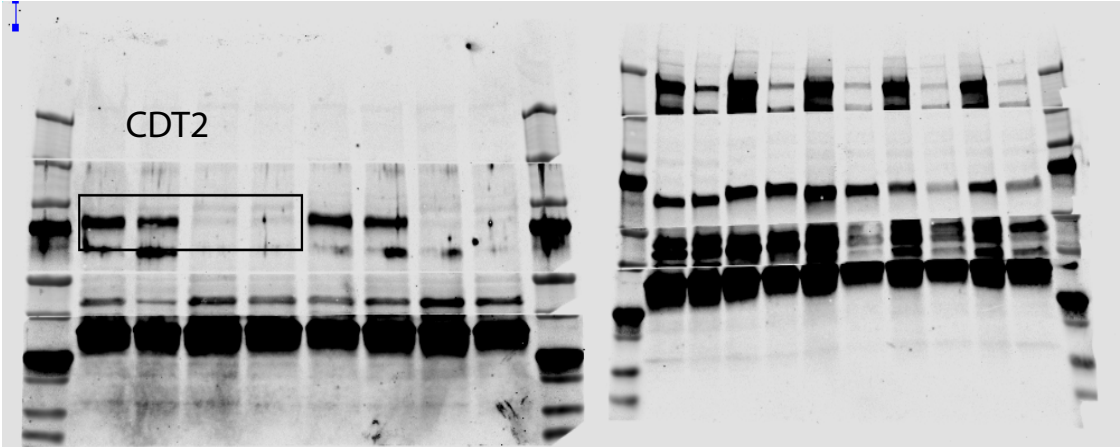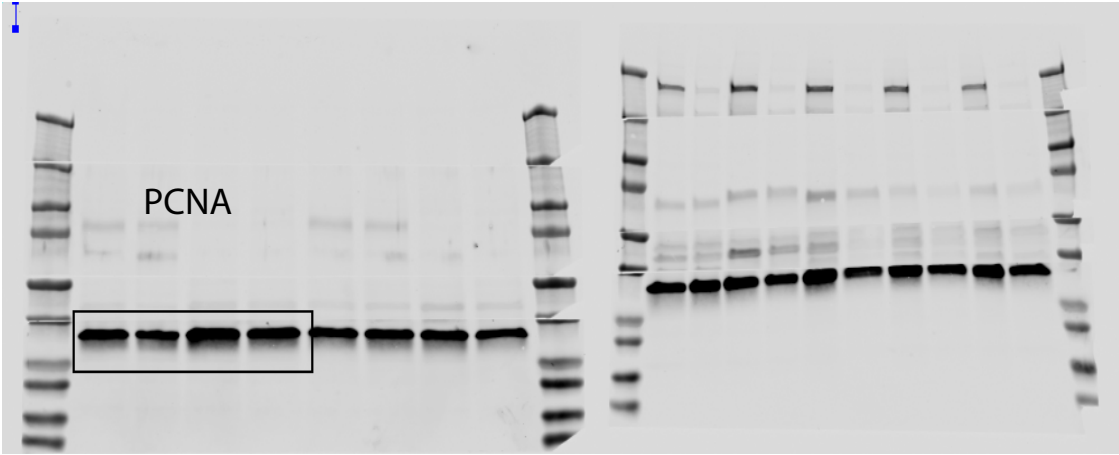

Supplement: Supplementary file 7 [file LSA-2023-02230_SdataFS3.1.pdf]
